# Temporal Evolution of Drug Resistance to HIV Integrase Inhibitors

**DOI:** 10.3390/v18050540

**Published:** 2026-05-08

**Authors:** Indrani Choudhuri, Jocelyn G. Olvera, Avik Biswas, Allan Haldane, Ronald M. Levy, Dmitry Lyumkis

**Affiliations:** 1 The Salk Institute for Biological Studies, La Jolla, CA 92037, USA; ichoudhuri@salk.edu (I.C.); jolvera@salk.edu (J.G.O.);; 2 Molecular Biology Section, Division of Biological Sciences, University of California San Diego, La Jolla, CA 92093, USA; 3 Department of Physics, University of California San Diego, La Jolla, CA 92093, USA; 4 Department of Physics, Temple University, Philadelphia, PA 19122, USA; 5 Department of Chemistry, Temple University, Philadelphia, PA 19122, USA

**Keywords:** drug resistance, antiretroviral therapy, epistasis, resistance evolution, integrase, strand transfer inhibitors, viral fitness

## Abstract

HIV-1 integrase (IN) strand transfer inhibitors (INSTIs) are central to modern antiretroviral therapy (ART) because of their high potency and durable effect on viral suppression. However, drug resistance mutations (DRMs) within HIV-1 IN emerge, which can compromise long-term treatment efficacy. Many distinct DRMs that arise under INSTI therapy have been extensively tabulated in public repositories and literature. However, the timelines over which they emerge, accumulate, and consolidate in patients have not been systematically integrated across clinical and experimental studies. In this review, we synthesize current evidence on the temporal evolution of DRMs within HIV-1 IN by examining mutational kinetic data from viruses derived from people living with HIV/AIDS (PLWH) and from in vitro selection experiments. We compare experimental timelines to recent computational predictions derived from Potts-based fitness landscapes coupled with kinetic Monte Carlo simulations and identify reproducible kinetic classes that distinguish fast-, intermediate-, and slow-emerging DRMs. Rapidly emerging DRMs such as E92Q and N155H typically appear early under drug pressure and often represent low-barrier adaptive responses, whereas the most clinically consequential mutations, such as Q148H/K/R, G140A/S, and E138K, arise only after extended therapy and generally require compensatory mutational backgrounds to persist. Although absolute emergence times vary substantially between in vivo and in vitro systems, consistent temporal trends across datasets support the existence of underlying epistatic constraints that shape drug resistance evolution. Understanding DRM timelines is clinically relevant because it provides a framework for interpreting resistance detected at virological failure, informs optimal timing of resistance testing, and may enable earlier identification of high-risk evolutionary trajectories before durable resistance is established.

## 1. Introduction

More than four decades into the human immunodeficiency virus (HIV) epidemic, advances in antiretroviral therapy (ART) have redefined what it means to live with the virus. Treatment, prevention, and public health education initiatives have contributed to a gradual decline in new infections, especially in regions with strong healthcare infrastructure. However, with over 40 million people still living with HIV (PLWH) and more than a million new infections each year, viral infection remains a significant global health concern. Global disparities also remain in both access to treatment and the ability to monitor and respond to emerging drug resistance [1].

HIV targets CD4+ T cells, progressively weakening the immune system and leading to immune dysregulation that compromises the body’s ability to fight infections. Without treatment, this can result in severe immunodeficiency, marking the onset of acquired immunodeficiency syndrome (AIDS), a condition characterized by opportunistic infections and high morbidity and mortality [2]. Fortunately, the increased availability of ART has transformed HIV infection into a manageable chronic condition for individuals receiving consistent treatment, significantly improving both life expectancy and quality of life. ART regimens target specific stages of the HIV replication cycle and currently rely on seven distinct drug classes that enable effective combination therapy, which suppress viral load and reduce the risk of resistance by limiting the virus’s ability to mutate and evade treatment [3,4].

The integrase (IN) strand transfer inhibitors (INSTIs) represent current first-line compounds in ART regimens. INSTIs stand out for their high potency [5]. There are five INSTIs that are currently approved for clinical use by the United States Food and Drug Administration (FDA). These include the first-generation drugs Raltegravir (RAL, approved in 2007) and Elvitegravir (EVG, approved in 2012), as well as the second-generation drugs Dolutegravir (DTG, approved in 2013), Bictegravir (BIC, approved in 2018), and Cabotegravir (CAB, approved in 2022). While RAL and EVG have a relatively low genetic barrier to resistance, DTG and BIC demonstrate a notably higher barrier, with CAB exhibiting an intermediate profile [5]. INSTIs block the strand transfer step during HIV integration, thereby preventing insertion of viral DNA (vDNA) into the host DNA. Strand transfer is carried out by the nucleoprotein complex formed by multimers of HIV IN bound to the linear ends of vDNA, referred to as an intasome [6,7,8]. INSTIs engage the active site of IN in the context of the intasome, forming bonds with both the enzyme and vDNA ends [6,9,10,11]. All INSTIs share a core structural motif with a planar arrangement of heteroatoms that chelate two Mg^2+^ ions at the IN catalytic site [6,10,12]. They also contain an aromatic side chain that interacts with vDNA [6,10,12]. This configuration displaces the viral 3’-end from the active site and effectively blocks the strand transfer reaction [6].

The emergence of drug-resistant mutations (DRMs) in HIV IN compromises the efficacy of INSTIs. Like other DRMs, INSTI resistance mutations can be broadly categorized as primary or accessory [13]. Primary DRMs directly reduce drug susceptibility and can arise alone or alongside accessory mutations. Accessory mutations can either augment resistance to drug or serve as compensatory mutations that help offset the replication fitness costs associated with primary DRMs. Both effects facilitate the emergence of major drug resistance pathways [13]. IN mutations such as Y143C/R, N155H, and Q148H/K/R were often observed in patients experiencing virological failure to first-generation INSTIs [14,15,16]. The second-generation INSTIs DTG and BIC demonstrate improved binding kinetics and broader activity against resistant viral strains, but resistance to these drugs still occurs, particularly in treatment-experienced individuals or in settings with adherence challenges [5,17]. Clinically observed DRMs such as R263K, G118R, and Q148H/K/R, often accompanied by accessory/compensatory mutations, have been associated with reduced susceptibility to both DTG and BIC. Combinations of INSTI resistance mutations involving primary DRMs generally confer greater resistance to second-generation INSTIs than single substitution alone, particularly in pathways center on Q148H/K/R, G118R, or R263K, where additional accessory changes can substantially amplify resistance to dolutegravir, bictegravir, and cabotegravir [18,19,20,21,22,23]. These and other mutations are increasingly documented in clinical settings and represent a growing concern in certain populations [24,25,26].

Cabotegravir (CAB) has recently emerged as the first long-acting injectable drug for treating PLWH. CAB extends the utility of INSTIs through monthly or bimonthly dosing regimens and offers benefits for adherence and stigma reduction [27,28,29]. Long-acting cabotegravir (CAB-LA) is also approved for use in pre-exposure prophylaxis (PrEP), where it has shown efficacy in preventing HIV acquisition in high-risk populations [30,31,32]. A Phase 2b single-arm study (NCT06741397), currently overseen by ViiV Healthcare, is evaluating the safety of a new CAB formulation designed to enable dosing every four months [33]. Although resistance data specific to CAB remains limited, in vitro studies using patient-derived viral isolates suggest that CAB may be more susceptible to several frequently occurring DRMs than DTG and BIC, with resistance emerging more readily under CAB pressure [20,34,35]. Recent work further highlights that mutations such as E138K and those in the Q148H/K/R pathway can reduce CAB susceptibility, and that some mutational combinations may have a greater impact on CAB than other second-generation INSTIs [36]. As with other antiretroviral therapies, the risk of resistance is shaped by multiple factors, including patient adherence, treatment history, pharmacokinetics, and viral characteristics. Multiple excellent reviews have comprehensively examined INSTI resistance, discussing mechanisms of DRM action [17,37], cataloging DRMs associated with various treatments [24,25,26,38,39,40,41], and defining relevant pharmacological considerations [42,43]. However, the timeframe over which specific DRMs arise in a viral population, an important consideration for understanding viral evolution, has received little attention in the literature and represents a key knowledge gap in the field. This review aims to fill that gap by beginning to address the following questions:

(1) What do current studies reveal about the timing and progression of DRMs during INSTI treatment?
(2) Are there specific DRMs that consistently emerge early or late during therapy?
(3) What sequence-dependent patterns underlie the differential timing of fast- versus slow-emerging DRMs in patients?

While we focus on INSTIs in this work, our broader intent is for this review to serve as a motivator to study, report upon, and define the temporal dynamics of drug resistance evolution in HIV. The relatively limited resistance to INSTIs, in comparison to other classes of inhibitors targeting reverse transcriptase (RT) or protease (PR), makes a survey of temporal dynamics manageable. However, similar observational analyses can readily be extended to inhibitors of RT and PR, and in the future, capsid (CA). By integrating data from computational predictions, clinical (in vivo) DRM emergence data, and laboratory-based (in vitro) selection experiments, we hope to motivate the study and systematic reporting of temporal resistance trajectories in HIV. Our objective is for this work to provide a framework for both future research and clinical applications, particularly in the context of personalized therapies.

### 1.1. Kinetic Coevolutionary Models Predict Temporal Emergence of HIV Drug Resistance

A recent report followed the time course of 52 DRMs in the viral enzymes PR, RT, and IN, the primary targets of antiretroviral therapy (ART) [44]. The DRMs were all selected based on the criterion that they appear with at least 1% frequency in PLWH (subtype B) and are also classified as primary/major DRMs in the Stanford HIVDB (Table 1). Building upon prior computational advances [45,46,47,48], this study used a Potts sequence-covariation statistical-energy model built from multiple sequence alignments (MSAs) of HIV protein sequences derived from drug-experienced PLWH. Operationally, the Potts model is a maximum entropy model constructed to capture the single-site and pairwise amino-acid frequencies observed in the MSA. The goal of Potts inference is to estimate the direct couplings between sites and specifically to determine conditional dependencies rather than raw correlations that can arise indirectly through chains of interactions across many residues. Thus, the Potts model is designed to capture the epistatic constraint networks that shape which mutational combinations are viable under drug pressure.

The Potts model provides a global mapping from sequence to statistical energy such that sequences that are more prevalent in the MSA have, on average, more favorable (lower) energies. Potts energy is often used as a proxy for the fitness of a sequence [47]. The change in Potts energy due to a mutation thereby measures the fitness change caused by a mutation relative to the wild-type residue. Changes in Potts energy have been shown to correspond well to different experimental measures of fitness [49,50,51,52]. Importantly, the Potts energy function includes pairwise coupling terms between the position of interest and all other sites, meaning that the energetic effect of a mutation at one position depends on the residues that are present at other positions. Thus, background dependence is a direct consequence of the epistatic interactions in the protein [47].

The Potts fitness landscape on its own tells us which sequences are favored under drug selection pressure but does not directly provide the kinetics of the acquisition of mutations. To obtain the kinetics of the acquisition of drug resistance, we combined a drug-experienced Potts fitness model with a Kinetic Monte Carlo (KMC) procedure to generate sequence trajectories starting from an ensemble of drug-naïve patient sequences [44]. The KMC procedure generates evolutionary trajectories as a sequence of proposed point mutations: at each step, a residue substitution is proposed (a stand-in for a newly arising mutant in a host), and it is accepted or rejected using a Metropolis-type rule that depends on the Potts energy change caused by that substitution. Accepted moves correspond to substitutions that fix in the within-host consensus due to improved fitness under drug pressure; rejected moves correspond to mutants that arise but fail to fix. Time is tracked in units of attempted mutations per position, and characteristic “acquisition times” for each DRM can be estimated by fitting the rise in its frequency across many simulated trajectories.

The results (Table 1) demonstrated that certain DRMs appeared rapidly following drug exposure and exhibit a high “adaptive frequency”, or pre-emptive likelihood to emerge in drug-naive sequences soon after the application of drug pressure due to a lack of epistatic constraint. In contrast, other DRMs are epistatically contingent on the emergence of accessory mutations that appear only after prolonged selective drug pressure. The predicted time constants from our KMC simulations closely aligned with clinical timelines of acquisition of DRMs in the literature, supporting the role of sequence-dependent epistatic interactions in shaping the kinetics of resistance evolution. Crucially, this computational framework is not trained on the order of mutation appearance. The Potts model is inferred only from cross-sectional sequence prevalence patterns in drug-experienced HIV patients (between host sequence datasets) and does not ingest longitudinal patient histories or explicit temporal mutation orderings. The apparent ordering emerges when trajectories start from drug-naïve sequences and evolve under a drug-experienced fitness landscape. In this framework, DRMs that are already compatible with typical drug-naïve backgrounds arise quickly, whereas DRMs that are penalized unless compensatory mutations are present arise more slowly. In [44], this distinction is formalized via background-dependent measures such as the sequence-specific propensity (“adaptive frequency”) of a DRM to arise at different likelihoods that is dependent on the background, and how additional mutations form an “epistatic barrier” to its acquisition.

**Figure 1:**
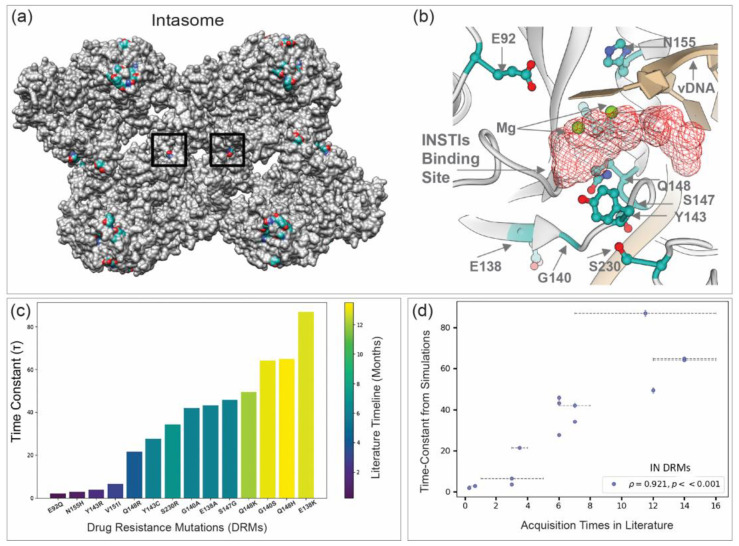
(**a**) The HIV-1 intasome (PDB: 9BW9) is displayed as a surface. The major INSTI DRMs are highlighted in cyan and the two active sites where drugs bind are indicated by black square boxes. (**b**) A close-up view of one of the active sites, illustrating the positions of the major INSTI DRMs. (**c**) The previously reported correspondence between literature-reported acquisition times of major HIV-1 IN DRMs under INSTI pressure, and the time constants (τ) obtained from KMC simulations. The height of each bar represents the simulated time constant, while the color scale indicates the emergence time of each DRM in patient populations, as reported in the literature. (**d**) The Spearman rank-order correlation between KMC-derived time constants and literature acquisition times for IN DRMs. Grey dashed lines along the abscissa denote the range of acquisition times reported in the literature when multiple studies are available, while those along the ordinate represent the uncertainty in the computational time constants obtained from best-fit curves [38].

This work motivated a re-examination of the temporal evolution of clinical INSTI resistance, incorporating newly available data and comparisons with in vitro selection studies. To limit scope, we focused our report on resistance mutations in HIV-1 integrase (IN). At the time of publication of the original report [44], there were thirteen DRMs in HIV-1 IN that met the criteria defined above: E92Q, N155H, Y143R, Q148R, Y143C, S230R, G140A, E138A, S147G, Q148K, G140S, Q148H, E138K. These thirteen clinically relevant DRMs confer intermediate to high-level resistance to commonly used INSTIs. The location of the residue positions that become DRMs is shown on the structure of the HIV intasome (Figure 1a) and displayed within the intasome active site (there are two identical active sites per intasome) where INSTIs bind (Figure 1b). Each of these 13 frequently occurring DRMs resides in proximity to the bound INSTI ligand. The computed characteristic acquisition times from KMC simulations are reported in units of attempted mutations per position and can be binned into fast, intermediate, and slow classes: ~1–10 for fast, ~10–24 for intermediate, and >24 for slow DRMs. The clinical timelines were divided into three categories based on their acquisition times: fast (0–3 months), intermediate (4–6 months), and slow (more than 6 months). In the original study, predicted time constants for IN closely matched acquisition rates reported in the literature under drug pressure (Figure 1c,d).

Additional IN DRMs have since been reported in viral sequences from PLWH but were excluded from the original models because they either did not appear with >1% frequency and/or were underrepresented in the drug-experienced MSA. These include mutations such as M50I, H51Y, T66A/I/K, L74M, G118R, and R263K, which arise more commonly in viral sequences derived from PLWH failing second-generation INSTIs. Because these specific DRMs are becoming increasingly important as second-generation INSTIs are administered more broadly, they will be discussed toward the end of this review.

The ability of KMC simulations integrated with Potts fitness landscapes to recapitulate major clinical DRM emergence patterns reinforces the idea that drug resistance is shaped by the epistatic fitness landscape of HIV-1 IN. The implication is that rapidly acquired DRMs begin accumulating immediately upon drug pressure, whereas slowly acquired DRMs are contingent on the prior accumulation of accessory/compensatory mutations that relax epistatic constraints and restore fitness. This alignment between predicted and observed timelines suggests that computational frameworks can provide an early-warning system for which mutations are most likely to emerge under clinical drug pressure. However, such models cannot fully capture patient-level complexity, including adherence, pharmacokinetics, viral reservoirs, and host immune pressure. Thus, we were motivated to more carefully examine the predictive modeling alongside clinical and experimental observations.

### 1.2. Data Curation for Experimental Emergence of IN DRMs in PLWH and Through Cell Culture Selections

Clinical research has documented the emergence of major HIV-1 IN DRMs in PLWH [24,25,38,53]. These in vivo studies track viral evolution in ART-treated individuals, capturing the dynamics of resistance acquisition under the influence of complex parameters, such as host immunity, adherence patterns, and pharmacokinetics, factors that are absent in in vitro systems. By sequencing viral populations longitudinally, these studies can provide insight into the order of DRM emergence, as well as mutational pathways that can lead to virologic failure.

A complementary approach to clinical observations is to perform in vitro selection experiments of DRM evolution [54]. In vitro selections expose virus to escalating concentrations of drug(s) under controlled cell culture conditions, typically beginning with a single molecular viral clone. By removing confounding external factors (viral diversity, immune response, patient adherence, drug pharmacokinetics, etc.), such experiments provide a simpler and more controllable setting through which to isolate the virus’s intrinsic capacity to evolve under selective pressure, based on the internal viral fitness landscape. However, in vitro selection experiments also depend on numerous experimental conditions, such as the cell lines used for viral passaging, the exact drug that is used, the dose escalation protocol, passaging intervals, experimental setup, among other factors, also leading to variability.

We curated timelines for the emergence of DRMs both in vivo and in vitro. To curate in vivo timelines, we sought to compile peer-reviewed studies reporting viral sequencing data from PLWH as comprehensively as possible within the scope of this review, focusing on studies where INSTI DRMs were detected at defined time points or with sufficient longitudinal sampling to infer the timing of emergence. We prioritized studies with comparable cohorts and regular sequencing intervals. We note that in many clinical in vivo studies, DRMs may only be reported at the end of the trial, and regular sequencing is not performed. Given the focus on the temporal evolution of DRMs, we do not report on trials in which the time-course of DRM emergence is left out. Although we aimed for broad coverage, inadvertent omissions may remain given the broad scope of this review. The studies included in Table 2, Table 3, Table 4 and Table 5 span publications from 2008 to 2026 and report DRMs that emerge under first- and second-generation INSTI regimens. Studies reporting only summarized mutation frequencies or prevalence estimates were excluded because they did not provide individual-level temporal detail required for inclusion in our mutation timeline tables. We found that most DRM emergence timelines were reported during the clinical trial period but not after the broader administration of the drug following its approval. Examples of studies that report summarized mutation frequencies include but are not limited to the following references [55,56,57,58,59].

**Table 2:** In vivo timeline of IN DRM emergence in PLWH undergoing ART.

Focus DRMs	Additional Mutations	Population *	Methodology of Sequencing	Past Treatment History	Total Subjects	INSTI	Timeline for Focus DRMs (~Weeks)	Timeline for Addition al DRMs (~Weeks)	Reference
E92Q	T66K, N155H/S, E138K, S147G, Q148R	Gilead 183-0105 study patients & routine clinical samples	RT-nested PCR, Sanger Sequencing (21 clones/ timepoint) ^3^	Heavily ART-experienced	16	EVG or RAL	2	2–48	[60]
	E157Q	Single Case Report	Genotype resistance testing ^2^	ART-experienced	1	EVG; BIC (E157Q)	39	91	[61]
N155H	T66K, E92Q, E138K, S147G, Q148R	Gilead 183-0105 study patients & routine clinical samples	RT-nested PCR, Sanger Sequencing (21 clones/ timepoint) ^3^	Heavily ART-experienced	16	EVG and RAL	2	2–48	[60]
	E92Q, T97A, E138K, G140S, Q148R/H, Y143R	SCOPE, BENCHMRK (Phase III), & Monogram	GeneSeq (dye-terminator), PhenoSense Integrase Assay ^1^	ART-experienced, INSTI-naive	200	RAL	8	8–24	[62]
	T66I, G118R, E138K, R263K; H51Y, E92Q, T97A	VICONEL (Cohort Study)	Next-Generation Sequencing ^3^	ART-experienced	85	DTG	56	84–160	[63]
	R263K	VICONEL (Cohort Study)	Next-Generation Sequencing ^3^	ART-experienced	85	DTG	148	148	[63]
Y143R	E92Q, T97A, E138K, G140S, Q148R/H N155H	SCOPE, BENCHMRK (Phase III), & Monogram	GeneSeq (dye-terminator), PhenoSense Integrase Assay ^1^	ART-experienced, INSTI-naive	200	RAL	16–28	8–24	[62]
	T97A, L74M, G163R, N155H, S230R, V72I, V201I	ANRS CO3 Aquitaine Cohort	RT–PCR, nested PCR, direct (population) Sanger sequencing (Beckman CEQ 2000 XL) ^1^	ART-experienced	51 (11 VF)	RAL	12–24	4–24	[64]
Q148R	E92Q, T97A, E138K, Y143R G140A/S, N155H	SCOPE, BENCHMRK (Phase III), & Monogram	GeneSeq (dye-terminator), PhenoSense Integrase Assay ^1^	ART-experienced, INSTI-naive	200	RAL	11–16	8–24	[62]
	S147G, N155H	DOLAM	Trugene, Illumina MiSeq ultra-deep sequencing ^3^	ART-experienced	91 (31 triple, 29 DTG/3TC, 31 DTG mono)	DTG	24	24	[65]
	E138K, G140A	MONODOLU	Sanger, Illumina MiSeq ultra-deep sequencing ^1^	ART-experienced	28	DTG	24	24–30	[66]
	A128T	SeroPrEP study, routine care (USA)	Single-genome sequencing (SGS) ^1^	PrEP-only (CAB-LA); INSTI naive	1	CAB	4	4	[67]
	L74I	PrEP trial HPTN 083 (Phase 3 randomized)	Single-genome sequencing + GenoSure PRIme ^1^	PrEP-only (CAB-LA); INSTI-naive	34	CAB	8	8	[68]
	—	PrEP trial HPTN 083 (Phase 3 randomized)	Single-genome sequencing + GenoSure PRIme ^1^	PrEP-only (CAB-LA); INSTI-naive	34	CAB	4	—	[68]
Y143C	T97A, E138A, L74M, Y143G/R	Clinic samples, RAL-failure	RT-nested PCR, Sanger sequencing (ABI PRISM 3100) ^1^	ART-experienced	11 (10 VF)	RAL	24	24–64	[69]
S230R	—	DOMONO Phase II	Sanger Sequencing ^1^	ART-experienced	104	DTG	30	--	[70]
E138A	T97A, G140S, Q148H, L74M, V75L, S147G, G149A, N155H	VIKING-4 Phase III Clinical Trial	GeneSeq, PhenoSense (Monogram), initial genotyping by Quest Diagnostics ^1^	Heavily ART-experienced, INSTI resistance	30	DTG	2–24	2–48	[71]
G140A	T97A, E138K, S147G, N155H,	VIKING-4 Phase III Clinical Trial	GeneSeq, PhenoSense (Monogram), initial genotyping by Quest Diagnostics ^1^	Heavily ART-experienced, INSTI resistance	30	DTG	24	2–48	[71]
	E138K; Q148R	Clinical Study (MONODOLU)	Sanger + Illumina MiSeq ultra-deep sequencing ^1^	ART-experienced	28	DTG	24	24–30	[66]
S147G	T97A, Q148R/H, E138K, N155H, G140A	VIKING-4 Phase III Clinical Trial	GeneSeq, PhenoSense (Monogram), initial genotyping by Quest Diagnostics ^1^	Heavily ART-experienced, INSTI resistance	30	DTG	24–48	2–48	[71]
	Q148R, N155H	DOLAM	Trugene, Illumina MiSeq ultra-deep sequencing ^3^	ART-experienced	91 (31 triple, 29 DTG/3TC, 31 DTG mono)	DTG	24	24	[65]
	N155H	MONCAY	Sanger Sequencing ^2^	ART-experienced, INSTI-naive	158	DTG	36	36–136	[72]
	M50V, E138T, L74M, R263K, A49G, V201I/V	IMPAACT P1093	Sanger Sequencing ^1^	ART-experienced. Resistance to NRTI/NNRT/PI	142 (36 VF)	DTG	136	36–136	[73]
Q148K	G140S	SCOPE (Cohort Study)	GeneSeq Integrase (Sanger-based), PhenoSense Integrase Assay (Monogram) ^1^	ART-experienced	29 VF	RAL and EVG	20	19–63	[74]
	N155H, Q148H/R, Y143C/R, E92Q	BENCHMRK-1 NCT00293267 BENCHMRK-2 NCT00293254	Sanger Sequencing ^1^	ART experienced	699	RAL	48	16–48	[75]
G140S	E138K, N155H	DOLAM	Trugene, Illumina MiSeq ultra-deep sequencing ^3^	ART-experienced	91 (31 triple, 29 DTG/3TC, 31 DTG mono)	DTG	24	24	[65]
	Q148H	DOLUMONO	Sanger Sequencing ^1^	ART-experienced	31	DTG	24	24	[76]
	Q148H	Clinic routine care (Northwestern Poland cohort)	ViroSeq 2.8 (BigDye/ABI 3500) ^1^	ART-experienced, ART-naive	80 (12 VF)	RAL	65	10–65	[77]
	Q148H, N155H, T97A, D232N, E138K, G163R	PROGRESS Study	ViroSeq and GeneSeq ^1^	ART-naive	206	RAL	64	16–96	[78]
	Q148H/R/K, Y143R/C/H E138A	VIKING Trial Phase II (ING112961)	PhenoSense Integrase Assay (Monogram) + GenSeq ^1^	ART-experienced	51 (27 RAL failure, 24 RAL resistance)	RAL	24	4–24	[79]
Q148H	N155H, G140S, Y143R, E92Q, T97A,	SCOPE, BENCHMRK (Phase III), & Monogram	GeneSeq (dye-terminator), PhenoSense Integrase Assay ^1^	ART-experienced, INSTI-naive	200	RAL	18–24	8–24	[62]
	G140S	SCOPE (Cohort Study)	GeneSeq Integrase (Sanger-based), PhenoSense Integrase Assay (Monogram) ^1^	ART-experienced	29 VF	RAL and EVG	20	19–63	[74]
	G140S	Clinic routine care (Northwestern Poland cohort)	ViroSeq 2.8 (BigDye/ABI 3500) ^1^	ART-experienced, ART-Naive	80 (12 VF)	RAL	16–51	10–65	[77]
	T97A, E138T, G140S	Case Series NIH Protocol (NCT01976715)	Sanger + NGS (Illumina); PhenoSense ^1^	Extensively ART experienced	2	DTG	24	8–24	[80]
	G140S	DOLUMONO (Cohort Study)	Sanger Sequencing ^1^	ART-experienced	31	DTG	24	4–24	[76]
	G140S	Clinic routine care (Northwestern Poland cohort)	ViroSeq 2.8 (BigDye/ABI 3500) ^1^	ART-experienced, ART-naive	80 (12 VF)	RAL	65	10–65	[77]
	G140S, N155H, T97A, D232N, E138K, G163R	PROGRESS Study	ViroSeq and GeneSeq ^1^	ART-naive	206	RAL	64	16–96	[78]
	G140S	VIKING Trial Phase II (ING112961)	PhenoSense Integrase Assay (Monogram) + GenSeq ^1^	ART-experienced	51 (27 RAL failure, 24 RAL resistance)	RAL	24	4–24	[79]
E138K	T97A, Q148R/H, S147G, N155H, G140A	VIKING-4 Phase III Clinical Trial	GeneSeq, PhenoSense (Monogram), initial genotyping by Quest Diagnostics ^1^	Heavily ART-experienced, INSTI resistance	30	DTG	24	2–48	[71]
	E92Q, T97A, S147G, G140A/C/S, M50I, H51Y, L74M, R263K	WAVES study GS-US-236-0128	Deep Sequencing ^1^	ART-naïve	39	EVG	48	48	[81]
	Q148H, N155H, T97A, D232N, E138K, G163R	PROGRESS Study	ViroSeq and GeneSeq ^1^	ART-naive	206	RAL	64	16–96	[78]
	T66A, T97A, G118R	VICONEL (Cohort Study)	Next-Generation Sequencing ^3^	ART-experienced	85	DTG	120	120	[63]
	T66I, G118R, R263K	VICONEL (Cohort Study)	Next-Generation Sequencing ^3^	ART-experienced	85	DTG	112	112–152	[63]

* The patient population for the clinical study mentioned in Table 2 is defined according to the NCI Dictionary of Cancer Terms (https://www.cancer.gov/publications/dictionaries/cancer-terms/search/case/?searchMode=Begins; accessed on 31 March 2025). ^1^ Sequenced region: Integrase (IN); ^2^ Sequenced regions: Reverse Transcriptase (RT) and Integrase (IN); ^3^ Sequenced regions: Protease (PR), Reverse Transcriptase (RT), and Integrase (IN).

To curate in vitro timelines, we compiled studies reporting the emergence of major INSTI DRMs in cell culture experiments. For simplicity, we focused on experiments using reference strains (e.g., NL4-3), wild-type viral subtypes, or patient-derived isolates that were absent from major INSTI DRMs in the integrase coding sequence. For each study, we extracted the earliest reported time at which the mutation was detected (reported in weeks or passages). The results are summarized in Table 2 for in vivo timelines and Table 3 for in vitro timelines.

**Table 3:** Timeline of emergence of major IN DRMs in vitro.

Mutations	Additional Mutations	Cell Line	Viral Strain	INSTI	INSTI Concentration (nM)	Primary Mutation Initial Detection (Weeks) *	Additional Mutations Detection Range (Weeks)	Reference
E92Q	T66I; D10E, T66I A128A, Q148R	MT-4	HIV-1 IIIB	EVG	5–10,000	4	2–14	[82]
	N155H/R263K	MT-2	NL4-3	EVG	6.4–800	6	4–8	[83]
	—	MT-2	NL4-3	DTG	6.4	8	—	[83]
	T124A; Q148K; M154I	MT-2	HIV-1 IIIB	RAL	0.256–4000	8	2–10	[84]
N155H	E92Q, E92Q/R263K	MT-2	NL4-3	EVG	6.4–800	4	6–8	[83]
	V151, I204T; E92Q/M154I	MT-2	HIV-1 IIIB	RAL	0.59–360	4	2–12	[85]
	—	MT-2	NL4-3	RAL	32–160	4	—	[83]
	Q95K, S230R *Baseline polymorphisms* ^3^	CBMCs	Patient Isolate Subtype B	EVG	1–10,000	12	12–24	[86]
	— *Baseline polymorphisms* ^1^	CBMCs	Recombinant (pt-derived IN into NL4-3)	CAB	1–100	36–38	—	[34]
	T124A/V151I; Q148R; I204T	MT-2	HIV-1 IIIB	RAL	6.4–4000	4	2–10	[84]
Y143R	L74M, V151I *Baseline polymorphisms* ^7^	CBMCs	Recombinant (pt-derived IN into NL4-3)	RAL	1–10,000	36–38	36–38	[34]
	T97A, V151I *Baseline polymorphisms* ^8^	CBMCs	Recombinant (pt-derived IN into NL4-3)	RAL	1–10,000	36–38	36–38	[34]
	N155H, P233S *Baseline polymorphisms* ^11^	SupT1	Recombinant (pt-derived Subtype B into HXB2)	RAL	2–1024	4–5	4–5	[87]
Q148R	A128A/T, E92Q, T66I, D10E, H114Y, A128T	MT-4	HIV-1 IIIB	EVG	5–10,000	11	2–20	[82]
	E138K	MT-2	NL4-3	EVG	6.4–160	4	8	[83]
	—	MT-2	NL4-3	EVG	32–160	2	—	[83]
	S147G *Baseline polymorphisms* ^6^	CBMCs	Patient Isolate Subtype B	EVG	1–10,000	24	8	[86]
	—	MT-2	NL4-3	RAL	6.4–32	6	—	[83]
	E138K, G140GS, L74I, Q95K *Baseline polymorphisms* ^2^	CBMCs	Recombinant (pt-derived IN into NL4-3)	CAB	1–250	18	18–38	[34]
	L74M, E138K, R263K *Baseline polymorphisms* ^5^	CBMCs	Patient Isolate Subtype AG	CAB	1–500	16	8–46	[34]
	Q95R, S147G *Baseline polymorphisms* ^1^	CBMCs	Recombinant (pt-derived IN into NL4-3)	EVG	1–2500	36–38	36–38	[34]
	T66I	MT-2	NL4-3	EVG	0.7–90	4	4–12	[88]
Y143C	N155H, L74M, G163R, S230K/R/N *Baseline polymorphisms* ^4^	SupT1	Recombinant (pt-derived Subtype B into HXB2)	RAL	2–1024	4–5	4–5	[87]
S230R	Q95K, N155H *Baseline polymorphisms* ^3^	CBMCs	Patient Isolate Subtype B	EVG	1–10,000	12	12–30	[86]
	T66I, Q146R *Baseline polymorphisms* ^1^	CBMCs	Recombinant (pt-derived IN into NL4-3)	EVG	1–1000	36–38	36–38	[34]
	T66I, Q95K, E157Q, *Baseline polymorphisms* ^5^	CBMCs	Recombinant (pt-derived IN into NL4-3)	EVG	1–2500	36	18–38	[34]
G140A	Q148R, E138K, V151IV *Baseline polymorphisms* ^9^	CBMCs	Patient Isolate Subtype D	RAL	1–10,000	36	18–38	[86]
	Q148K	SupT1	HXB2	RAL	2–1024	4–5	4–5	[34]
	Q148K, G147G/S; Q148K, S147G, L74M, T97AT *Baseline polymorphisms* ^2^	CBMCs	Patient Isolate Subtype B	CAB	0.1–1000	24–30	8–46	[34]
S147G	Q148R *Baseline polymorphisms* ^6^	CBMCs	Patient Isolate Subtype B	EVG	10–10,000	8	24	[86]
	T66I, G163GR, Q146R, Q95R *Baseline polymorphisms* ^10^	CBMCs	Patient Isolate Subtype AG	EVG	1–1000	16	8–46	[34]
	H51Y *Baseline polymorphisms* ^1^	CBMCs	Recombinant (pt-derived IN into NL4-3)	DTG	5–25	36–38	36–38	[34]
	Q148K, S153FS, G140S, L74M, T97AT *Baseline polymorphisms* ^2^	CBMCs	Patient Isolate Subtype B	CAB	1–1000	24–30	8–30	[34]
Q148K	G140A, E138K	SupT1	HXB2	RAL	2–1024	4–5	4–5	[87]
	E138K, G140C *Baseline polymorphisms* ^4^	SupT1	Recombinant (pt-derived Subtype B into HXB2)	RAL	2–1024	4 –5	4–5	[87]
	N155H, I204T, Q148R, E138K, G140S, V151I, N17S, G163R, G140C, E92Q, M154I	MT-2	HIV-1IIIB	RAL	0.26–4000	2	2–10	[89]
G140S	S153FS, Q148K, T97AT, S147GS, L74M *Baseline polymorphisms* ^2^	CBMCs	Patient Isolate Subtype B	CAB	1–1000	28	8–46	[34]
	Q148R, E138K, L74I *Baseline polymorphisms* ^5^	CBMCs	Recombinant (pt-derived IN into NL4-3)	CAB	2.5–2500	36	18–36	[34]
	Q148R, V151I/N155H	MT-2	HIV-1IIIB	RAL	0.59–360	10	10–12	[85]
	Q148R, A265A; Q148H *Baseline polymorphisms* ^4^	SupT1	Recombinant (pt-derived Subtype B into HXB2)	RAL	2–1024	4–5	4–5	[87]
Q148H	G140S, G163R; N232H, G140S, C280Y *Baseline polymorphisms* ^11^	SupT1	Recombinant (pt-derived Subtype B into HXB2)	RAL	2–1024	4–5	4–5	[87]
	G140S *Baseline polymorphisms* ^4^	SupT1	Recombinant (pt-derived Subtype B into HXB2)	RAL	2–1024	4–5	4–5	[87]
E138K	L74M, Q148R, R263K *Baseline polymorphisms* ^2^	CBMCs	Patient Isolate AG	CAB	1–500	16	8–46	[34]
	Q148R	MT-2	NL4-3	EVG	6.4–160	8	8	[83]
	G140C, Q148K *Baseline polymorphisms* ^4^	SupT1	Recombinant (pt-derived Subtype B into HXB2)	RAL	2–1024	4–5	4–5	[87]

* Indicates the first observed timepoint for the primary mutation under escalating drug pressure; ^1^ D10E, E11D, S24N, V31IV, V32I, L45IL, V72I, L101I, T112A, S119PRST, T122IT, G123S, A124AT, R127K, K136KN, V201IV, N232D; ^2^ K7R, D10E, E11D, K14R, V31I, V32I, M50L, V72I, L101I, G123S, A124N, R127K, S195T, I203M, I220L, Y227F, N232D; ^3^ E11D, R20K, V31I, S39N, M50I, S119T, T124N, G193E, V201I, D286N; ^4^ V72I, N232D, A265V; ^5^ D10AE, E11D, V37I, K111A, T112A, S119P, G123S, A124T, T125A, R127K, V201IV, T206S, I208IL, N232D; ^6^ S17N, S24D, D25E, S39C, V201I, S255Q; ^7^ D10E, V31I, L68LV, V72I, I73IV, T112IT, I113V, G123S, R127K, I162IV, V201I, N232D, R284GR; ^8^ D10E, V72I, L101I, G123S, A124T, R127K, I203M, T206ST, N232D; ^9^ D10E, S17N, K34KR, L45I, V72I, L101I, T112IV, G123S, T125A, R127K, E157Q, K160E, D167E, G193E, V201I, I220V, N232D, L234I, D288N; ^10^ D10E, K14R, V31I, V72I, L101I, T112V, G123S, T125A, R127K, G134N, I135V, K136T, V201I, T206S, N232D, L234I, D256E, R269K, S283G; ^11^ V72I, N232D, A265V.

### 1.3. Temporal Dynamics of DRMs In Vivo and In Vitro

In this section, we discuss each of the 13 individual DRMs and summarize the temporal dynamics of their emergence both in vivo and in vitro. These mutations primarily arise in response to first-generation INSTIs, but some also arise in response to second-generation INSTIs. In the case of the latter, the genetic background of circulating viruses in INSTI-experienced PLWH likely predisposes the evolution of certain DRMs. Because there is also more data available for patients failing first-generation INSTI therapy, the computational Potts models on which the selections are based are more robust for delineating the time-course of DRMs emerging in response to first-generation INSTIs. The individual DRMs are discussed in order, from fast to slow, according to τ values derived from Potts analyses and shown in Figure 1c. We follow the framework established in prior computational work [44] to assign DRMs into three categories for both in vivo and in vitro temporal dynamics:

1. Fast—DRMs emerge within ≤12 weeks (~3 months) of treatment.
2. Intermediate—DRMs emerge between 12–24 weeks (3–6 months).
3. Slow—DRMs emerge after ≥24 weeks (6 months) or more.

E92Q: E92Q is detected at low prevalence (~3.2%) among INSTI-experienced PLWH and is classified as an early-emerging DRM under EVG and RAL therapy based on computational analyses. Longitudinal in vivo studies have reported the appearance of E92Q within approximately two weeks of virologic failure [60], placing it among the most rapidly emerging IN DRMs, consistent with its classification from Potts evolutionary analysis. In vitro selection experiments identify E92Q within ten weeks under EVG or dolutegravir (DTG) pressure across multiple cell lines [82,83]. Clinically, E92Q is frequently observed in early resistance profiles and often in association with other primary INSTI DRMs, including N155H, supporting its role as an early adaptive response to INSTI exposure [60,90]. E92Q is most closely associated with the N155H pathway and is frequently observed as an accessory mutation in INSTI-resistant clinical isolates, particularly following EVG or RAL exposure [60,90,91]. In a recent single-case report, E92Q was first detected during EVG exposure, whereas E157Q was subsequently detected after the regimen was switched to BIC, extending the in vivo timeline of resistance pathway across sequential INSTI exposures [61].

N155H: N155H is a well-described major IN DRM and an early resistance pathway under INSTI pressure, particularly under EVG and RAL and detected in up to 31% of INSTI-treated PLWH [60,62]. Longitudinal in vivo studies report that N155H can emerge within approximately two weeks [60], consistent with its computational classification as a fast-emerging DRM. However, N155H is often evolutionarily unstable. In RAL-treated individuals, N155H frequently appears early but is later displaced by other DRMs that confer greater resistance and improved viral replicative capacity, such as Q148H or Y143R [62]. More recent in vivo data also document N155H during DTG exposure, although reports with detailed temporal timelines remain limited [63]. In vitro selection experiments similarly show early emergence of N155H, typically within ~four weeks under RAL or EVG pressure, although timelines vary with experimental conditions [83,85]. Notably, when using subtype B virus derived from patient isolates containing background polymorphisms, N155H emerged more slowly, around 12 weeks, suggesting an intermediate timeline in that context [86]. N155H may be a fast-emerging DRM, albeit one that represents a transitional step in the evolution toward high-level drug resistance that leads to INSTI treatment failure.

Y143R: Y143R is an RAL-associated IN DRM observed at moderate prevalence (~5.5%) among INSTI-experienced individuals. In vivo, Y143R typically emerges at later time-points and less frequently than N155H, appearing 12–28 weeks after the onset of treatment [62,64], consistent with its intermediate kinetic classification in Potts evolutionary analysis. In vitro, however, Y143R has demonstrated variable selection timelines depending on the system used. In one study using recombinant viruses in cord blood mononuclear cells (CBMCs), Y143R appeared only after prolonged RAL exposure (~36–38 weeks) alongside L74M, V151I, and T97A, consistent with a slow and selective emergence trajectory [34]. By contrast, another study using recombinant patient-derived virus in SupT1 cells detected Y143R within ~4–5 weeks, co-emerging with N155H and other mutations under similar RAL pressure [87]. Once established, Y143R is often maintained as a dominant DRM, highlighting its role as a stable evolutionary endpoint within the RAL resistance pathway. Y143R evolution appears to be specific to RAL presumably due to the pi/pi interaction between Tyr143 and oxadiazole ring of RAL, which is disrupted by the Y143R mutation [92].

Q148R: Q148R is a key IN DRM conferring high-level resistance to first-generation INSTIs, detected at approximately 5% prevalence among INSTI-experienced individuals, particularly in advanced treatment failure. In vivo, Q148R typically emerges after early DRMs like N155H. Longitudinal clinical studies further demonstrate that, once established, Q148R is frequently associated alongside G140A/S or E138K [62,65], which serve as compensatory mutations to account for fitness defects imparted by Q148R [93]. More recently, in vivo Q148R has also been documented in CAB-LA breakthrough infection, with detection within ~4–8 weeks in HPTN 083 cases, indicating that this pathway is not restricted to conventional treatment-failure settings [68]. In vitro selection studies report variable emergence timelines for Q148R, ranging from 4 to 38 weeks, depending on the experimental context [34,83,85,86,88]. Under EVG pressure, Q148R emerges as early as week 2–4 in MT-2 cells without and with E138K as an additional mutation, respectively [83]. However, in CBMCs using patient-derived sequences, Q148R detection is delayed until weeks 18–46 and occurs alongside multiple accessory mutations such as S147G, E138K, G140S, L74M, and R263K [34,86]. These findings underscore the context-dependent kinetics of Q148R evolution in vitro.

Y143C: Y143C is a raltegravir (RAL)-associated IN DRM observed at low prevalence (~2.4%) among INSTI-experienced individuals. In clinical settings, Y143C emerges at later time-points than both N155H and Y143R, appearing several weeks to months after treatment onset (~24–64 weeks) [69], consistent with its intermediate kinetic classification via Potts analysis. These findings suggest that Y143C primarily contributes to INSTI resistance during the later stages of treatment failure, rather than serving as an early adaptive mutation. By contrast, in vitro selection experiments using SupT1 cells and recombinant patient-derived virus under RAL pressure have reported earlier detection of Y143C (~4–5 weeks), and alongside N155H, L74M, G163R, S230K/R/N, N232D, and A265V [87]. Like Y143R, the mutation Y143C is specific to RAL, although it appears to be less likely to persist over time by comparison.

S230R: S230R is an infrequently observed IN DRM, detected at low prevalence (~4%) among INSTI-experienced individuals. It is rarely selected as an isolated mutation and typically arises in multi-mutation sequence backgrounds, particularly under dolutegravir (DTG) pressure. In vivo, S230R has been reported as a late-emerging mutation [70]. In the DOMONO trial, S230R was absent at baseline and detected only at virologic failure during dolutegravir monotherapy, emerging approximately 24–48 weeks after treatment switch, consistent with late selection under prolonged DTG pressure rather than rapid early adaptation [70]. By contrast, in vitro selection experiments under EVG pressure reported earlier emergence, with S230R appearing within 12 to 36 weeks [34,86]. The slower kinetic profile in vivo may be related to S230R arising within complex resistance pathways and influenced pressure.

G140A: G140A is a less commonly observed accessory IN DRM, detected at low prevalence (~1.1%) among INSTI-experienced individuals, most commonly in viruses harboring Q148K/R (but not Q148H) DRMs and often co-occurring with E138K. In vivo, G140A typically emerges after the selection of primary Q148K/R mutations, reflecting an accessory role in resistance. The MONODOLU clinical study reported G140A emergence approximately 24 weeks after treatment initiation alongside E138K and Q148R, which was followed by virological rebound [66]. Rhee et al. classifies G140A as an accessory IN DRM that emerges alongside Q148H/R/K substitutions in late treatment phase, functioning primarily as a compensatory mutation in vivo [24]. In vitro selection experiments support delayed emergence, with G140A detected only after prolonged INSTI exposure (~36–38 weeks), along with E138K, Q148R, and V151I substitutions [86]. The frequent co-occurrence of G140A with E138K and Q148K/R may enhance viral replicative capacity and resistance levels, explaining why G140A-containing sequences persist under long-term INSTI pressure rather than appearing as early or independent DRMs.

E138A: E138A is an accessory IN DRM detected at low prevalence (~1.7%) among INSTI-experienced individuals and is rarely observed as an isolated resistance determinant. E138A was detected as a pre-existing accessory mutation in INSTI-resistant PLWH participating in the VIIKING-4 Phase III Clinical trial [71]. The timeframe for the development of this mutation was observed during dolutegravir functional monotherapy, with a wide range of emergence timelines (2–24 weeks) [71]. This mutation frequently occurs alongside the common DRMs G140A/S and Q148H/K/R, serving to partially restore viral infectivity in resistant backgrounds [94]. To date, no in vitro selections have reported the emergence of E138A as a primary mutation under INSTI pressure [54]. This mutation is also less prevalent than its more abundant E138K counterpart.

S147G: S147G is an infrequently observed IN substitution, with low prevalence (~1%) among INSTI-experienced individuals, and is rarely detected as an isolated resistance determinant. S147G has been reported late or at low frequency in clinical studies, including VIKING-4, DOLAM, and MONCAY, all after ~6–9 months on therapy and within complex drug-resistant sequence backgrounds [65,71,72]. In vitro selection experiments using CAB demonstrated emergence of S147G between 24–30 weeks of passaging [34]. Using EVG, the mutation emerged at week ~16 in one study [34], and as fast as week 8 in another study [86]. Longitudinal clinical observations indicate that S147G does not function as a primary DRM, but instead accumulates gradually under sustained drug pressure, consistent with its slower kinetic behavior and limited independent contribution to INSTI resistance.

Q148K: Q148K is the least frequently observed substitution among DRMs at IN position 148, detected at low prevalence (~1%) among INSTI-experienced individuals (Table 1). In vivo, data from the BENCHMRK trials show that Q148K was absent at baseline and detected only after RAL treatment failure, typically in combination with accessory mutations, indicating its emergence after prolonged (~48 weeks) drug exposure [75]. Clinical observations further indicate that Q148K is most often accompanied by accessory mutations such as E138K and G140A, which are thought to partially compensate for the substantial fitness cost imposed by Q148 substitutions [25,93,95]. By contrast, in vitro selection experiments under RAL pressure have demonstrated faster emergence kinetics, with Q148K detected as early as two to four weeks of continuous exposure [87,89]. The computational model classifies Q148K as an intermediate mutation (on the slower side), which is more consistent with in vivo observations.

G140S: G140S is one of the most widely studied IN DRMs, detected at high prevalence (~20%) among INSTI-experienced individuals and is most frequently observed in viruses harboring Q148H and E138K DRMs. The G140S mutation is one of the first DRMs to have been identified in PLWH under INSTI therapy, the other being Q148H [14,60]. In addition to co-occurring with Q148H, G140S also frequently arises with Q148K and Q148R [62,75]. In vivo, G140S occurs nearly universally along with or shortly after the appearance of Q148H mutations, becoming detectable months (24–65 weeks) following therapy onset [65,76,77,78,79]. Early in vitro selection experiments demonstrated the emergence of G140S after 10-weeks and in combination with Q148R [85]. However, more recent in vitro selection experiments with clinical isolates, HIV subtype B, and under CAB pressure demonstrated delayed kinetics, with G140S arising only after prolonged exposure to drug, but also in combination with Q148K/R [34]. G140S has been selected in combination with Q148H in vitro, but only when selections were initiated with viruses already harboring the Q148H DRM [83,96]. The frequent co-selection of G140S with mutations Q148H/R and late appearance across both clinical and experimental systems suggests that G140S contributes to resistance as an accessory mutation.

Q148H: Q148H is a clinically significant DRM within the Q148 resistance pathway, detected at high prevalence (~18.6%) among INSTI-experienced individuals. The Q148H mutation is one of the most widely recognized IN DRMs, leading to rapid failure of first-generation drugs and conferring modest levels of resistance to second-generation drugs [24,71]. In vivo, Q148H most commonly emerges alongside G140S in both clinical trials and case studies around 16–65 weeks [62,65,74,76,77,78,79,80]. Interestingly, Q148H only appeared in a single in vitro selection study, arising during passaging of a viral isolate extracted and cloned from a patient undergoing INSTI-based ART [87]. Q148H does not typically emerge within in vitro selection experiments [54], possibly due to limited sampling or insufficient timeframes for selection experiments, but also due to differences between in vivo and in vitro settings. We note, however, that since Q148H is a well-known DRM that is selected in vivo, several studies initiated passaging with viruses already harboring the Q148H mutation [84,96]. This may further reflect the relevance of Q148H as one of the latest DRMs, appearing only after prolonged therapy. These experimental findings are supportive of computational predictions suggesting that Q148H is one of the latest emerging DRMs.

E138K: E138K is an accessory IN DRM detected at low prevalence (~3.4%) among INSTI-experienced individuals. It is primarily observed in association with Q148H/K/R substitutions. In vivo, E138K typically emerges in concert with primary resistance mutations, appearing ~24–64 weeks in patients [71,78,81]. In vitro selection studies, E138K is detected between 4–16 weeks across multiple cell lines and drug pressures, often alongside Q148R [34,83,87]. Selection data, combined with single-round infectivity analyses, suggest that E138K may play a compensatory role, stabilizing and consolidating Q148-based resistance profiles [97]. Both in vitro and in vivo observations indicate that E138K does not initiate resistance pathways but instead strengthens resistant phenotypes once primary Q148H/K/R mutations are established. The computational model classifies E138K as the last emerging DRM, but there is a broad distribution of timescales observed under different experimental observations.

**Table 4:** Timeline of emergence of G118R and R263K in vivo.

Focus DRMs	Additional Mutations	Population *	Methodology of Sequencing	Past Treatment History	Total Subjects	INSTI	Timeline for Focus DRMs (~Weeks)	Timeline for Additional DRMs (~Weeks)	Reference
G118R	H51Y, E138K, R263K (Patient 1, subtype C); D67N-RT (Patient 2, subtype B)	DAWNING (phase IIIb)	PhenoSense, GenoSure (Monogram) ^3^	ART-experienced, INSTI-naive, PI-naive	624 (312 DTG)	DTG	48–52	48–52	[98]
	H51Y, T66I, L74I, E138K, Q148R, R263K	DAWNING (phase IIIb)	GeneSeq, PhenoSense (Monogram) ^3^	ART-experienced, INSTI-naive	314 (DTG arm)	DTG	36–168	36–168	[99]
	—	ACTG A5381–Hakim (Prospective cohort)	Population-based sequencing ^2^	ART-experienced	173	DTG	24	—	[100]
	L74M, V75A,	IMPAACT P1093 (Pediatric participant 2)	Clonal IN genotyping, phenotyping (Monogram) ^3^	ART-experienced, INSTI-naive	8	DTG	192	192	[73]
	*Baseline polymorphism: V151I*	IMPAACT P1093 (Pediatric participant 3)	Clonal IN genotyping, phenotyping (Monogram) ^3^	ART-experienced, INSTI-naive	8	DTG	52	52	[73]
	E138K, G163R, T66A	ARTIST trial (phase II)	Sanger sequencing ^3^	ART-experienced, INSTI-naive	192	DTG	146	146	[101]
	—	ARTIST trial (phase II)	Sanger sequencing ^3^	ART-experienced, INSTI-naive	192	DTG	96	—	[101]
R263K	A49G, M50V, E138T, S147G, V201I *Baseline polymorphism: L74V*	IMPAACT P1093 (Pediatric participant 1)	Clonal IN genotyping, phenotyping (Monogram) ^3^	ART-experienced, INSTI-naive	8	DTG	36	36–136	[73]
	—	Single Case Report	Genotypic resistance testing ^2^	ART-experienced	1	DTG	104	—	[102]
	N155H	VICONEL (Cohort Study)	Next-Generation Sequencing ^3^	ART-experienced	85	DTG	148	148	[63]
	G118R	RESINA study, single case report (Germany)	NGS (Illumina MiSeq), ultradeep sequencing ^2^	ART-naïve (first-line)	1	DTG	8	15	[103]
	S153F	Montréal HIV Primo Infection Cohort (Canada)	Near full-genome ultradeep sequencing (Illumina MiSeq) ^1^	ART-naïve (first-line)	1	DTG	8	12	[104]
	E157Q **	Case Series (Hospital De Egas Moniz)	Sanger Sequencing ^3^	ART-experienced	2	DTG	4	4	[105]
	—	Case Series (Hospital De Egas Moniz)	Sanger Sequencing ^3^	ART-experienced	2	DTG	12	—	[105]
	—	GEMINI-1 and GEMINI-2 pooled, multicenter Phase III trials	Sequencing Genotypic and phenotypic resistance testing (Monogram, PhenoSense) ^2^	ART-naïve (first-line)	1433 (716 DTG/3TC; 717 DTG/TDF/FTC)	DTG	144	—	[106]
	E157Q **	Single Case Report (Hospital Universitario de Burgos)_	Sanger Sequencing ^3^	Heavily ART-experienced, INSTI-experienced	1	BIC	36	36	[107]
	M50I	PrEP trial HPTN 083 (Phase 3 randomized)	Single-genome sequencing + GenoSure PRIme ^1^	PrEP-only (CAB-LA); INSTI-naive	34	CAB	3	3	[68]
	—	ACTG A5381–Hakim (Prospective cohort)	Population-based sequencing ^2^	ART-experienced	173	DTG	24	—	[100]
	—	ACTG A5353 (Phase 2 Pilot Study)	Population-based genotyping ^3^	ART-naïve, treatment-naive	120	DTG	14	—	[108]

* The patient population for the clinical study mentioned in Table 2 is defined according to the NCI Dictionary of Cancer. Terms (www.cancer.gov/publications/dictionaries/cancer-terms/search/case/?searchMode=Begins; accessed on 31 March 2025)); ** E157Q is a polymorphic substitution. At the time of sequencing, it was already present alongside R263K. ^1^ Sequenced region: Integrase (IN); ^2^ Sequenced regions: Reverse Transcriptase (RT) and Integrase (IN); ^3^ Se-quenced regions: Protease (PR), Reverse Transcriptase (RT), and Integrase (IN).

**Table 5:** Timeline of emergence of G118R and R263K in vitro.

Mutations	Additional Mutations	Cell Line	Viral Strain	INSTI	INSTI Concentration (nM)	Primary Mutation Initial Detection (Weeks) *	Additional Mutations Detection Range (Weeks)	Reference
G118R	E138K, T66A	CBMCs	Patient Isolate Subtype C	DTG	0.1–1000	29	50–66	[109]
	E69E/K Baseline polymorphism ^1^	CBMCs	Patient Isolate Subtype AG	DTG	25–50	20	20–37	[23]
	H51Y Baseline polymorphisms ^2^	CBMCS	Patient Isolate C	DTG	50	20	37	[23]
R263K	Baseline polymorphisms ^3^	CBMCs	Patient Isolate Subtype AG	DTG	0.5–50	16	—	[34]
	Q148R, E138K, L74M, Baseline polymorphisms ^4^	CMBCs	Patient Isolate Subtype AG	CAB	0.5–500	8	16–46	[34]
	S153A Baseline polymorphism ^5^	CMBCs	Patient Isolate Subtype B	EVG	0.5–1000	24	24–46	[34]
	M50I	MT-2	NL4-3	BIC	10–500	7	22–33	[110]
	T66I	MT-2	NL4-3	EVG	Not reported	3	8–17	[110]
	S143Y, S119R, M50I	MT-2	NL4-3	DTG	10–120	12	12–29	[110]
	D229E, R224Q **	SupT1	NL4-3	DTG	0.1–2000	46	30–46	[111]
	Baseline polymorphism ^6^	CBMCs	Patient Isolate Subtype B	DTG	50	20	_—_	[23]
	E138K, W243G Baseline polymorphism ^7^	CBMCs	Patient Isolate Subtype B	DTG	50	20	20–37	[23]
	S153Y, R166K ** Baseline polymorphism ^8^	CBMCs	Patient Isolate Subtype B	DTG	50	20	20–37	[23]
	M50I, V151I Baseline polymorphism ^9^	CBMCs	NL4-3	DTG	50	20	20–37	[23]
	D288E Baseline polymorphism ^10^	CBMCs	Patient Isolate Subtype B	DTG	10–25	20	20–36	[23]

* Indicates the first observed timepoint for the primary mutation under escalating drug pressure; ** R263K transient in strain 5326, present as R263K/R mixture at Week 20, replaced by S153Y by Week 37; ^1^ V72I, T125A, V201I; ^2^ V72I, Q95P, T125A, V201I, I203M; ^3^ D10E, K14R, V31I, V72I, L101I, T112V, G123S, T125A, R127K, G134N, I135V, K136T, V201I, T206S, N232D, L234I, D256E, R269K, S283G; ^4^ D10E, K14R, V31I, V72I, L101I, T112V, G123S, T125A, R127K, G134N, I135V, K136T, V201I, T206S, N232D, L234I, D256E, R269K, S283G; ^5^ K7R, D10E, E11D, K14R, V31I, V32I, M50L, V72I, L101I, G123S, A124N, R127K, S195T, I203M, I220L, Y227F, N232D; ^6^ I72V; ^7^ M154I, V201I; ^8^ V72I, I203M; ^9^ I72V, I113V, L234V; ^10^ I203M.

### 1.4. Emergence of Additional DRMs Within HIV-1 IN

The DRMs discussed above are present at >1% frequency in PLWH and were classified as primary/major DRMs in the Stanford HIVDB that were explicitly analyzed in Biswas, Choudhury et al. [44]. However, with some exceptions, these DRMs are not as prominently selected in drug-naïve patient populations who are administered second-generation INSTIs as initiation therapy (DTG or BIC). There are additional relevant DRMs, especially those that arise in response to second-generation INSTIs, which include M50I, T66A/I/K, L74M, G118R, and R263K. The two most prominent DRMs are G118R and especially R263K, which are increasingly observed in PLWH who are administered second-generation INSTIs. Below, we briefly discuss the temporal evolution data on these DRMs. We bring specific attention to both G118R and R263K by tabulating in vivo and in vitro temporal evolution, respectively (Table 4 and Table 5).

M50I: Across clinical and experimental studies, M50I consistently appears as a late-emerging, accessory IN substitution. Clinical data from PLWH failing DTG-based therapy show that M50I rarely appears in isolation and is typically detected only in combination with primary mutations such as R263K, even after extended treatment exposure [112]. In vitro selection experiments recapitulate a delayed trajectory. Under sustained DTG pressure, R263K emerges first, followed by the gradual appearance of M50I at later passages at ~5 months [110]. These findings position M50I as an intermediate/late-emerging accessory mutation, which is likely coupled to the R263K DRM that arises under second-generation INSTI pressure.

T66A/I/K: Substitutions at IN residue 66, including T66I/A/K, are well established as early, primary elvitegravir (EVG) resistance mutations. In vivo, T66 variants can emerge as early as ~2 weeks following EVG initiation, often as single substitutions during early virologic failure, preceding or co-occurring with alternative DRMs such as E92Q, Q148H/K/R, or N155H [60]. In vitro, EVG selection experiments similarly demonstrate that T66I and T66A arise under low-to-moderate drug pressure, frequently preceding mutations such as R263K, F121Y, and S153Y across different cell systems [34,113]. Together, these findings support a role for T66 substitutions as low-barrier and early gateway mutations in the evolution of resistance.

L74M: L74M is an accessory IN substitution among INSTI-experienced individuals. In clinical studies, L74M has been reported early in treatment failure (8–24 weeks), often among the first accessory mutations to emerge, although detailed longitudinal data are limited [64,69,71]. In vitro selection studies offer more precise timelines, with L74M arising during early passages under INSTI pressure. L74M has been reported to co-emerge with primary mutations such as N155H (RAL) [85], Y143R (RAL), S147G and G140S (CAB), and E138K (CAB and RAL) [34], suggesting a broad accessory role across multiple resistance pathways.

G118R: G118R is a primary IN substitution that was initially observed at virologic failure in individuals receiving dolutegravir monotherapy [109]. Subsequently, G118R emerged more frequently after the widespread deployment of second-generation INSTIs. In INSTI-naïve patients enrolled in large phase III trials such as DAWNING, G118R was detected at virologic failure after prolonged drug exposure (~36–168 weeks) [98]. Pediatric data from IMPAACT P1093 documented emergence as late as 192 weeks [73]. A single first-line case report (RESINA study) demonstrated that G118R can emerge as early as 8 weeks under DTG pressure in ART-naïve individuals [103]. G118R tends to either emerge alone or in combination with other substitutions, most commonly R263K, H51Y, and/or E138K [103,112] and was associated with reduced viral replication capacity. In vitro selection experiments in CBMCs detected G118R emergence between 20 and 29 weeks depending on viral subtype and DTG concentration [23,109]. The emergence was typically accompanied by accessory mutations such as E138K, T66A, or H51Y, supporting a slow kinetic profile. These observations place G118R as a generally late-emerging, high-fitness-cost DRM associated with second-generation INSTIs, although early emergence in first-line settings has been documented [103].

R263K: R263K is a major second-generation INSTI-associated DRM most commonly reported under dolutegravir (DTG) pressure. Its early clinical appearance was reported in the SAILING trial and related DTG-based trials, where R263K was detected at virologic failure after prolonged DTG exposure [114]. Subsequent reports from DTG monotherapy or functional monotherapy settings further established R263K as a primary escape mutation capable of emerging in isolation in some patients, albeit with markedly reduced viral replication capacity [23,115]. Additional in vivo studies have since shown that R263K can emerge across a broad temporal range, from approximately 14–24 weeks in pilot and prospective studies [100,108], ~104 weeks in a single case report [102], and ~148 weeks in a cohort-based observation [63]. This broad range suggests that host factors, viral subtype, treatment history, regime activity, and adherence significantly influence the kinetics of R263K selection. In vitro selection experiments support these clinical observations: in MT-2 cells, R263K emerged after under BIC or DTG pressure, often alongside M50I, S119R, and S153Y [110]. In CBMC-based passage with patient-derived isolates, R263K was detected under DTG pressure, followed in some selectionsby the emergence of accessory mutations such as E138K and S153Y by weeks 20–37 [23,34]. Notably, under CAB pressure, R263K appeared as early as week 8 and was subsequently accompanied by the accumulation of Q148R pathway mutations (E138K, L74M) by week 46, indicating that R263K can coexist with and potentially facilitate the development of complex multi-mutation resistance profiles under second-generation INSTI pressure [34].

### 1.5. Emergence of DRMs Outside IN

Apart from mutations within IN, recent work indicates that INSTI resistance can involve mutations in viral genes outside IN, including Envelope (Env) and Nucleocapsid (NC) [111,116,117]. Earlier work from the Freed group reported that mutations in Env mutations can emerge under DTG selection pressure and reduce DTG susceptibility in vitro [116]. More recent in vitro selection experiments demonstrated that mutations in nucleocapsid (NC) also arise and contribute to INSTI resistance, particularly under prolonged selective pressure [117]. The timeline of mutation emergence reveals a sequential pattern. In selections conducted using NL4-3 virus under DTG pressure, Env mutations (Env-A541V) appeared at 19 weeks, followed by NC mutations (NC-N17S and G19S) and IN mutation (D229E) at 23 weeks. Primary IN mutations like IN-R263K appeared later and detected at low frequency only at 46 weeks [111]. In subtype C viruses (e.g., CH185), the NC and Env mutations emerged early (NC-A30T at week 6, and Env-A533V at week 8), preceding IN mutations that appeared much later (IN-R263K at week 20). Additional mutations continued to accumulate over extended passaging, including NC-D48N and Env-L764M at weeks 38 and 46 respectively [117].

Mutations outside the IN gene do not alter drug binding directly but instead act through indirect mechanisms. Mutations in Env enhance the ability of HIV to spread via cell-cell transmission and reduce susceptibility to multiple classes of antiretrovirals, includingINSTIs; when combined with target-gene resistance mutations, they can further increase resistance [118]. NC mutations accelerate early post-entry steps in the viral replication cycle and appear to be more specific to INSTIs, narrowing the window for these drugs to bind the intasome and block integration [117]. Mechanistically, NC-G19S shortened the time lag between reverse transcription completion and integration from ~5.5 h (wild-type) to ~2.4 h, with ~2.3-fold more integrated DNA than wild-type at 6 h post-infection [117]. In doing so, NC mutations enhance resistance conferred by IN mutations but do not rescue fitness. These findings highlight that INSTI resistance can be shaped by mutations outside the IN gene and suggest that additional, slowly accumulating changes may play a role under prolonged drug pressure. Clinically, these data point to the critical need for whole-genome sequencing of the virus to understand the full scope of resistance evolution, extending beyond the gene being targeted by ART.

### 1.6. General Trends and Insights from Computational and Experimental Data

Across computational and experimental approaches, a hierarchy for the temporal ordering of INSTI resistance evolution emerges. A small subset of fast-emerging DRMs appears early after drug exposure, typically within weeks to a few months, reflecting mutations that impose relatively modest fitness penalties and require minimal prior mutational context. These early trajectories are dominated by pathways selected under first-generation INSTIs, such as EVG and RAL (Table 2) and include mutations such as E92Q, N155H, and Y143C/R. By contrast, the most clinically consequential DRMs, particularly those compromising the efficacy of second-generation INSTIs, arise through intermediate and slow timeframes, presumably through multi-step evolutionary pathways (Table 2).

Slowly emerging DRMs may only become favorable after the virus has accumulated enough accessory mutations that reshape the fitness landscape and are often indicative of a complex evolutionary pathway shaped by epistatic constraints that limit their early selection. Within HIV-1 IN, these mutations are centered on substitutions at Q148 (H/K/R)—the Achilles’ heel for the INSTI drug class—with accessory mutations including G140A/S and E138A/K, the presence of which either restores viral fitness or accentuates high-level resistance (Table 1, Table 2 and Table 3). As a result, durable INSTI resistance rarely reflects a single mutational event, but instead emerges through evolutionary processes shaped by epistatic constraints.

**Figure 2:**
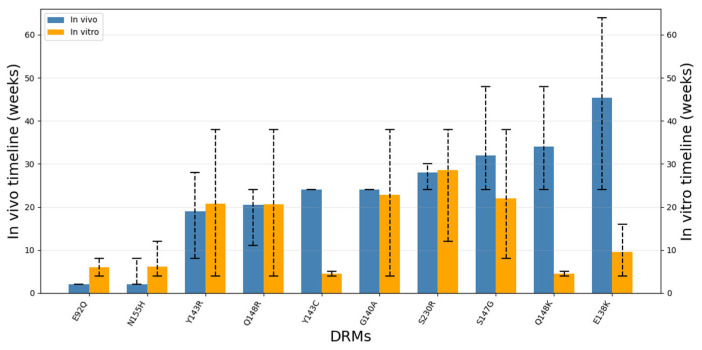
Comparison of in vivo and in vitro emergence timelines of clinically relevant HIV-1 integrase (IN) drug resistance mutations (DRMs), based on data summarized in Table 2 and Table 3. DRMs are ordered in ascending order according to their in vivo emergence time (Table 2). Both datasets are displayed using the same y-axis scale to enable direct visual comparison of the magnitude of the in vivo and in vitro emergence timelines. Black dashed lines denote the reported ranges of emergence times compiled from the literature. These ranges reflect variability across studies and are independent of specific INSTIs, drug concentrations, patient populations, experimental conditions, cell lines, sequence backgrounds, and other factors that may influence the emergence of DRMs.

Comparison of in vivo and in vitro timelines reveals that, despite limited sampling, the relative timing of DRMs may not be strictly conserved across experimental contexts (Figure 2). Mutations that arise rapidly in clinical settings do not uniformly do so under cell-culture selection, and conversely, substitutions that appear early under controlled in vitro drug pressure may emerge later in PLWH or require compensatory stabilization. These discrepancies indicate that resistance evolution does not follow a single universal trajectory but is instead strongly influenced by the biological and selective environment in which it occurs.

Despite the broad agreement across methodologies, discrepancies in absolute emergence times are evident (Table 2 and Table 3, Figure 2). Several DRMs that emerge rapidly in PLWH within ~2 weeks of virologic failure in clinical studies, most notably E92Q and N155H (Table 2 and Table 3), often appear later in cell-culture selections, where they may require many weeks of passaging and/or arise only under specific drug conditions. This discrepancy may reflect the influence of standing genetic variation, heterogeneous viral populations, and fluctuating pharmacokinetics in vivo, features that are absent within in vitro experiments that are initiated from clonally identical viral populations. Notably, E92Q and several other IN mutations occur within cytotoxic T lymphocyte epitopes, suggesting that host immune pressure may also contribute to their selection [119]. Conversely, mutations within the Q148 pathway (e.g., Q148K/H/R) are sometimes detected relatively early within in vitro experiments under sustained, high-intensity drug pressure using first-generation INSTIs, yet in vivo these substitutions typically emerge later and almost exclusively in combination with compensatory mutations such as G140A/S or E138K, underscoring the requirement for fitness stabilization for long-term persistence in PLWH. Additional discrepancies are observed for mutations such as Y143C, which can appear on slower timescales clinically [69] but at intermediate timeframes across some in vitro selection protocols [87], both under RAL pressure. These examples illustrate that differences between in vivo and in vitro timelines arise from a combination of biological factors (fitness costs, epistasis, and viral diversity) and methodological constraints (drug exposure regimens, sampling frequency, detection thresholds, viral replicative capacities, cellular permissiveness to infection, etc.). Accordingly, in vitro timelines are best interpreted as indicators of intrinsic mutational accessibility under controlled selection, while in vivo timelines reflect the integrated effects of viral fitness, host environment, adherence, and surveillance practices.

Potts-based statistical energy models, when coupled with kinetic Monte Carlo (KMC) simulations, recapitulate the relative ordering of fast-, intermediate-, and slow-emerging DRMs observed clinically, despite differences in absolute timescales. The KMC search is learned from real patient HIV sequences (the Potts model), such that proposed mutations are derived probabilistically from patient data that ensures the ensemble averages of the mutation frequencies are compatible with the viral fitness of these mutations in patient populations. In this regard, the current implementation of the KMC approach—by design—does not appear to be as well suited to simulate in vitro evolution, which likely contributes to deviations between simulated and clinical timelines and motivates future efforts to build separate models based on more comprehensive in vivo and in vitro datasets. However, the overall concordance between Potts-based predicted and experimental timelines supports the existence of an underlying fitness landscape that constrains resistance evolution. This indicates that computational predictions can be used as probabilistic forecasts of evolutionary accessibility that provide a framework to contextualize experimental and clinical observations, and to identify high-risk resistance trajectories before they become prevalent in PLWH.

## 2. Conclusions

The temporal evolution of DRMs in HIV-1 IN represents a complex interplay between viral genetics, host responses, and drug pharmacology. Motivated by new computational tools for predicting the dynamics of drug resistance evolution, we provide an integrated framework for understanding the temporal evolution of drug resistance within HIV-1 IN across clinical and experimental literature. Our goal is to shift focus from statically cataloging DRMs to understanding the dynamic processes by which these mutations arise, persist, and consolidate.

Like other viral drug targets, IN adapts under drug pressure by accumulating mutations in a manner that reflects trade-offs between drug resistance and viral fitness. Early mutations often confer modest resistance with minimal fitness cost, while later mutations typically lead to greater resistance but often arise with compensatory or accessory mutations that promote resistance or help restore fitness. Interrogating these evolutionary trajectories provides important context as to why certain DRMs appear quickly, while others take longer to accumulate. The temporal dynamics, including the timing, rate, and sequence of mutation acquisition, are essential to understanding how resistance evolves and may help guide pre-emptive clinical interventions. However, most studies report DRMs only after virological failure, without serial patient-level sampling and sequencing timepoints, offering little insight into when they may arise during treatment. Such studies were excluded when mutation-specific detection timepoints or intervals could not be extracted, including some recent studies of INSTI failure. This gap limits clinicians’ ability to detect emerging resistance early and adjust treatments before full virological failure occurs. If DRMs emerge in predictable intervals under certain treatment conditions, clinicians can optimize viral load monitoring schedules, implement early warning thresholds for resistance testing, and tailor interventions more effectively. Such insights would support more prognostic clinical decision-making for personalized therapy. Insight into the temporal dynamics of drug resistance evolution may also inform rational drug design aimed at preemptively addressing high-risk evolutionary pathways.

In the future, systematic temporal mapping of resistance evolution should become a routine component of both clinical trials and resistance surveillance, particularly as long-acting and high-barrier INSTIs (and other drugs) are deployed. Capturing the time when a mutation emerges can improve our ability to link viral evolution with patient outcomes. Discrepancies between in vivo and in vitro timelines underscore that no single experimental or modeling framework can fully capture the complexity of resistance evolution, highlighting the need to integrate diverse tools to understand resistance evolution. At the same time, novel computational models, such as Potts-based kinetic Monte Carlo simulations, offer scalable tools for forecasting resistance trajectories. More generally, integrating evolutionary modeling with clinical and experimental timelines provides a powerful framework for anticipating resistance trajectories, informing personalized treatment strategies, and guiding the design of next-generation antiretroviral therapies with improved durability.

## Figures and Tables

**Table 1:** The major DRMs in IN, prevalence, associated mutation list and timeline of emergence, the table is ordered by the prevalence of DRMs in ARV-treated patients.

Mutations ^1^	ARV-Treated Prevalence ^2^ (%)	Associated Mutations ^3^	Time Constant (τ) ± Error Using Potts Covariation Model ^4^	Timeline Class (Computational) ^3^
E92Q	3.2	N155H, E138K, S147G, Q148R	2.1 ± 0.106	Fast
N155H	24.3	L74M/F, G140A/S, Q148S/H/K	2.9 ± 0.100	Fast
Y143R	5.5	T97A, S230R	3.8 ± 0.132	Fast
Q148R	5.0	T97A, N155H, E138K, G140A/S	21.5 ± 0.496	Intermediate
Y143C	2.4	T97A	27.6 ± 0.415	Intermediate
S230R	4.0	Y143C	34.2 ± 0.477	Slow
E138A	1.7	Q148H/R/K	43.3 ± 0.820	Slow
G140A	1.1	E138K, Q148H/R/K, N155H	42.0 ± 1.227	Slow
S147G	1.0	N155H, Q148R, E138K	45.9 ± 1.087	Slow
Q148K	1.0	T97A, E138K, G140A/S, N155H	49.5 ± 1.323	Slow
G140S	20	E138K, Q148H/R/K, N155H	64.1 ± 0.137	Slow
Q148H	18.6	T97A, E138K, G140A/S, N155H	64.9 ± 0.192	Slow
E138K	3.4	G140A/S, Q148H/R/K,	86.9 ± 1.492	Slow

^1^ Major DRMs are selected based on their prevalence in ARV-treated patients. Table 1 lists the major DRMs, sorted by prevalence in ARV-treated PLWH of >1%; ^2^ Number of submitted sequences: 1220. We categorized DRMs as low prevalence (<5%), moderate prevalence (5–10%), and high prevalence (>10%) in drug-experienced patients living with HIV-1 subtype B. The numbers are based on prevalence data reported in Section S3 of the supplementary information from our previous work [38]; ^3^ Associated Mutations: Includes the major, accessory, and other classes of DRMs as categorized by Stanford HIVDB. (https://hivdb.stanford.edu/page/release-notes/; accessed on 10 January 2025); ^4^ The timeline of DRM emergence in patients undergoing ART, calculated using the Potts covariation model [44], is presented in this table.

## Data Availability

The original contributions presented in this study are included in this article. Further inquiries can be directed to the corresponding author.
